# Anodal transcranial direct current stimulation (atDCS) and functional transcranial Doppler sonography (fTCD) in healthy elderly and patients with MCI: modulation of age-related changes in word fluency and language lateralization

**DOI:** 10.3389/fragi.2023.1171133

**Published:** 2024-02-13

**Authors:** Florian Heimann, Sabine Weiss, Horst M. Müller

**Affiliations:** ^1^ Experimental Neurolinguistics Group, Bielefeld University, Bielefeld, Germany; ^2^ Center for Cognitive Interaction Technology (CITEC), Bielefeld University, Bielefeld, Germany; ^3^ Clinical Linguistics, Bielefeld University, Bielefeld, Germany

**Keywords:** transcranial direct current stimulation (tDCS), mild cognitive impairment (MCI), language lateralization, word fluency, functional transcranial Doppler sonography (FTCD)

## Abstract

**Introduction:** In addition to age-related changes in language, hemispheric lateralization of language functions steadily declines with age. Also, performance on word fluency tasks declines and is sensitive to the expression of dementia-related changes. The aim of this study is to evaluate the effect of anodal tDCS combined with a word fluency training on language lateralization and word fluency performance in healthy elderly subjects and in persons with mild cognitive impairment (MCI).

**Methods:** The effect of anodal tDCS over the left inferio frontal gyrus (IFG) was measured in a group of healthy elderly up to the age of 67 years (YG, Ø = 63.9 ± 3.02), a group of healthy elderly aged 68 years and older (OG, Ø = 78.1, ± 4.85), and a group of patients with MCI (Ø = 81.18, ± 7.35) by comparing performance in phonological and semantic word fluency tasks before and after 3 days of tDCS. Half of the experimental participants received sham stimulation. In addition, language lateralization was determined using a lateralization index (LI) measured with functional transcranial Doppler sonography (fTCD) before and after the stimulation period.

**Results:** Anodal tDCS was associated with significantly higher scores in phonological but not semantic word fluency in both YG and OG. In MCI patients, no difference was measured between the tDCS and sham groups in either word fluency task. fTCD showed significantly increased left lateralization in all three groups after the training phase. However, this effect was independent of tDCS and the degree of lateralization could not be predicted by word fluency performance in any of the groups.

**Discussion:** Phonological word fluency can be increased with atDCS in healthy elderly people by stimulating the IFG in a 3-day training. When cognitive decline has reached a certain stage, as is the case with MCI, this paradigm does not seem to be effective enough.

## 1 Introduction

In normal aging, many higher cognitive functions like language processing and speech can be affected by structural and functional deterioration of the brain. For example, both gray and white matter volume and its integrity decrease. Besides other processes, this leads to a decrease in inhibitory processes in the brain, which can also affect processing speed (Fraundorf et al., 2019). For instance, difficulties in naming objects can occur, or word retrieval in general may be impaired when producing sentences (Taler and Phillips, 2008; Burke and Shafto, 2011; Rinehardt et al., 2014). More complex language functions, such as those required in phonological and semantic word fluency (WF) tasks, involve fast and intact long-range neuronal networks. In phonological WF tasks, participants are asked to find as many words as possible that begin with a presented letter within a given time span. In semantic WF tasks, a semantic category (e.g., animals) is presented and participants have to find as many words as possible that correspond to this category. Both word fluency tasks place high demands on participants’ executive processes. On the one hand, they have to retrieve the appropriate items from memory and on the other hand, responses have to be initiated, previous responses have to be controlled, and inappropriate items have to be inhibited (Henry et al., 2004). In addition, these two tasks differ in terms of the strategies used to retrieve the words. Phonological WF requires the activation of lexical representations, whereas semantic WF relies on the retrieval of items that correspond to a higher-level concept. Semantic associations within the lexicon must be intact for the task to be completed successfully. These task differences are also reflected in the involvement of different brain regions. Recent fMRI findings suggest that phonological WF primarily activates (left) frontal brain regions, whereas semantic WF shows extensive activation in temporal and parietal networks (e.g., Baciu et al., 2016).

To compensate for declining performance in word fluency tasks, as well as in other cognitive tasks, certain compensatory mechanisms can be observed in older individuals compared to younger ones. One of these mechanisms discussed is *hemispheric asymmetry reduction in older adults* (HAROLD; Cabeza, 2002), which describes the change of formerly lateralized cognitive functions towards a more bihemispheric activity pattern. This correlates with a better performance of older persons in verbal-cognitive tasks. However, age-related functional changes in the healthy brain are exacerbated by neurodegenerative diseases such as Alzheimer’s dementia (AD) and compensatory mechanisms are reduced. Cognitive-linguistic impairments are already evident in the preliminary stages of dementia, referred to as mild cognitive impairment (MCI). Patients with MCI may show impairments in one or more different cognitive domains, such as episodic memory, verbal functions, visuospatial abilities, perceptual speed, and executive functions (Taler and Phillips, 2008; Petersen, 2016). Various cognitive tests are used to diagnose MCI (e.g., trail-making test, visuospatial tasks, word fluency tasks, naming and learning tasks). Among other things, a slowed reaction time and an increased error rate can be observed. In the domain of language, MCI patients often show problems in accessing semantic information, which manifests in word finding difficulties (Taler and Phillips, 2008).

An effective and widely used method for clinical detection of cognitive changes associated with MCI and dementia is the use of WF tasks described above. In general, patients with MCI perform better on these tasks than AD patients but worse than healthy controls (Murphy et al., 2006; Nutter-Upham et al., 2008; Weakley et al., 2013; Meinzer et al., 2015; Rajji, 2021). Scores on both phonological and semantic WF tasks can significantly predict the severity and mortality of later AD already at the MCI stage (Cerhan et al., 2002; Cosentino et al., 2006). In MCI, word fluency difficulties are more evident at the semantic level than at the phonological level (Chasles et al., 2020).

One reason for the differential importance of these word fluency tasks in diagnosis could be the involvement of different brain areas, as indicated by the underlying patterns of cerebral blood flow (CBF, Marcolini et al., 2022). CBF emerged as the most important predictor of performance on the word fluency tasks, with mean flow across all vessels important for semantic fluency, and left frontal flow the most important predictor of performance on phonological fluency (Keilp et al., 1999; Nutter-Upham et al., 2008; Vonk et al., 2020). The stronger association of semantic fluency with the most typical CBF deficits in AD, associated mainly with structural changes in temporal and parietal brain regions, argues for the higher sensitivity of this task in this disease. The deficits in phonological fluency, on the other hand, provide important information about the extent to which perfusion deficits have spread to the frontal cortex (Keilp et al., 1999; Vonk et al., 2020).

In the vast majority of people, language functions such as word retrieval in word fluency tasks are lateralized to one of the two hemispheres, mainly the left (Knecht et al., 2000). This can be observed with fMRI, but also with functional transcranial Doppler sonography (fTCD, Gutierrez-Sigut et al., 2015). The fTCD shows high correlations with lateralization patterns in fMRI (Deppe et al., 2004; Jansen et al., 2004; Hattemer et al., 2011; Somers et al., 2011) or the Wada test (Knecht et al., 1997). The advantage of this method is that it is well suited for larger samples or study designs with multiple sessions (Heimann et al., 2022). It is also particularly suitable for studying lateralization patterns in adults (Woodhead et al., 2019) regarding language functions and other cognitive functions, e.g., arithmetic (Connaughton et al., 2017; Woodhead et al., 2019) or spatial skills (Rosch et al., 2012). Its use in clinical populations, e.g., patients with epilepsy (Conradi et al., 2019) or Parkinson’s disease (Gutteridge et al., 2020), is also well studied. Since the mobile use of fTCD allows for free head movement and speaking, it is of even greater benefit in uncooperative patients, including patients suffering from dementia (Deppe et al., 2004).

The current study aims to detect the benefit of anodal tDCS over the left inferior frontal gyrus (IFG) when performing phonological and semantic WF tasks in patients with MCI and two groups of healthy controls. In recent decades, tDCS has been shown to be effective in research in healthy elderly (Meinzer et al., 2009; 2014; Cattaneo et al., 2011; Jeon and Han, 2012) and in the rehabilitation of patients with language and memory impairments in various neurological disorders (Fregni and Pascual-Leone, 2007). In healthy older adults, one session of anodal tDCS over the left ventral inferior frontal gyrus was associated with significant improvement in a semantic WF task (Birba et al., 2017). Participants even reached the level of younger controls, suggesting that a single session of anodal tDCS can temporarily reverse the nonbeneficial effects of aging on cognition, brain activity, and connectivity. Moreover, anodal tDCS over the left IFG significantly improved word retrieval performance in patients with MCI to the level of healthy controls (Birba et al., 2017). In other clinical studies, tDCS was associated with improved word retrieval performance in patients with aphasia (Meinzer et al., 2016), in patients with Parkinson’s disease (Manenti et al., 2016) and in neurological rehabilitation in patients with dementia (Holczer et al., 2020). Additionally, anodal tDCS intervention over the left frontotemporal cortex slowed down the progression of dementia symptoms and resulted in more physiological EEG patterns in AD (Gangemi et al., 2021). Other authors found that in patients with AD, abnormal patterns of EEG activity during memory processing were partially reversed by anodal tDCS and that this reversal correlated with an improvement in word recognition (Marceglia et al., 2016). Faster word recognition was found after one session of atDCS of the temporal cortex in elderly with MCI (Balduin-Philipps et al., 2021). Overall, however, conflicting or insufficient evidence was found for the efficacy of tDCS in dementia (Flöel, 2014; Chang et al., 2018; Buss et al., 2019), largely due to differences in study designs and stimulation parameters.

The aim of this study was to investigate phonological and semantic WF in healthy elderly individuals and individuals with MCI. This involved measuring language lateralization using fTCD before and after 3 days of WF tasks and concurrent atDCS/sham stimulation. Since most relevant studies have been based only on homogenous groups (e.g., only patients with specific diagnoses or groups of healthy elderly), we used this multi-dimensional approach. We compared three groups, including two healthy elderly groups (YG and OG) of two different age classes and one group of participants diagnosed with memory deficits (MCI). During the 3-day training period, participants received either anodal tDCS over the left IFG or sham stimulation. To our knowledge, no previous study has focused on the effect of tDCS on WF task performance and associated CBF changes over multiple days.

Therefore, in this study, we aimed to investigate the following questions:

1) Does anodal tDCS over the left IFG result in significant increase in phonological and/or semantic WF performance? Do the three groups benefit similarly?
2) Is there a difference in language lateralization during phonological and semantic WF tasks as measured by a language lateralization index (LI) using fTCD. Are there differences between groups?
3) Does the LI differ depending on whether participants were in the atDCS or sham group?
4) Are increases in WF performance associated with increases in CBF lateralization?

## 2 Materials and methods

### 2.1 Participants

31 native German speakers participated in the study (14 f, age range 60–100 years, Ø ±81.83, SD ± 10.25). The participants were all right-handed according to a modified version of the Edinburgh Handedness Inventory (Oldfield, 1971), while one participant reported a tendency towards left-handedness in early childhood but had been trained on the right hand. All had normal or corrected-to-normal hearing and vision. None of the participants had contraindications to tDCS according to the recommendations of Antal et al. (2017). Four participants had been taking plant-based medication for a diagnosed form of memory decline for more than 3 months without complaints, while all other participants were not taking any neuroactive medication.

The participants were either recruited through a study program for people over 60 at Bielefeld University, were volunteers who had read about the study in the local newspaper or in case of the MCI group, participants received a recommendation about the study from their treating neurologist. They came to the laboratory of Bielefeld University for the 5-day study or underwent the study at home for reasons of limited mobility. The participants were informed about the study orally as well as in written form and gave their written consent for participation and usage of data. After the diagnostic assessments in session 1, participants were ascribed to either the younger group of healthy participants (YG), the older group of healthy participants (OG), or the group of participants with MCI. The YG and OG each consisted of ten participants (5 atDCS, 5 sham), and the MCI group comprised 11 participants (6 atDCS, 5 sham).

After the last atDCS session, participants received an expense allowance for their participation. The study was approved by the Ethics Committee of Bielefeld University (Ethics Approval No. 2021-028) and was conducted according to the Declaration of Helsinki.

### 2.2 Experimental stimuli

The letters for the phonological WF task were derived from the ten most frequent German initial letters (defined by total amount of words beginning with the letters in question) according to the Wahrig-Brockhaus lexicon of the German Language (Wahrig-Burfeind et al., 2012). The five most frequent letters (A, K, H, B, S) were used for the diagnosis (session1) and the evaluation (session 5), and the remaining five (G, E, P, F, M) for the stimulation sessions. The items for the semantic WF task were chosen from a collection of semantic categories commonly used in German aphasia therapy. We used the categories *furniture*, *drinks*, *animals*, *electronic gadgets* and *diseases* for the diagnosis (session1) and the evaluation (session 5) and the categories *clothes*, *fruit*, *professions*, *vehicles,* and *sports* for the stimulation sessions. All semantic categories had to meet the criterion that a possible member of a category could not be assigned to any of the other categories.

### 2.3 Diagnostic session (session 1)

In order to compare healthy individuals and individuals with memory impairments, we first tested the participants for their cognitive ability with the DemTect (version A, Kalbe et al., 2004). We then grouped participants by age and memory performance. Those participants who scored less than the age-matched cut-off value of 12 on the DemTect and/or had a medically diagnosed memory/cognitive decline were ascribed to the MCI group (Ø = 81.2 years ± 7.8; EHI = 80.5 ± 19.6; DemTect = 10.6 ± 3.5); participants with a score higher than the age-matched cut-off value of 12 were ascribed to the healthy groups. Since the group of participants with memory complaints was older than the overall group of participants, we divided the group without memory complaints into a younger group of healthy adults (YG) with a maximum age of 67 years (Ø = 63.9 years ±3.0; EHI = 86.7 ± 20.5; DemTect = 17.7 ± 0.5) and an older group of healthy adults (OG) aged 68 years or older (Ø = 78.1 years ±5.4; EHI = 92.4 ± 8.7; DemTect = 16.6 ± 1.7). These three groups were compared for age, handedness and memory performance. In an ANOVA, the groups differed significantly in age (*F* (2,28) = 25.77, *p* ≤ .001) but not in handedness (*F* (2,28) = 1.3, *p* = .288). In a pairwise comparison using *t*-tests, only the younger group differed significantly in age from the MCI group (*t* (28) = −6.80, *p* ≤ 0.001) and the OG (*t* (28) = −5.46, *p* ≤ 0.001), while none of the older groups did (*t* (28) = −1.21, *p* = 0.46). The three groups differed significantly in their memory performance regarding their mean score on the DemTect (*F* (2,28) = 87.70, *p* ≤ 0.001). As expected, the group that scored less than the age norm on the memory test (MCI) had a significantly reduced memory performance than the group of healthy older participants (OG, >68 years) (*t* (28) = 10.31, *p* ≤ 0.001) and the healthy younger group (YG, 60–67 years) (*t* (28) = 12.22, *p* ≤ 0.001). There was no significant difference in memory performance between OG and YG (*t* (28) = 1.86, *p* = 0.17) although the younger group showed a tendency to perform better in the DemTect.

When assessing, participants had to perform word fluency (WF) tasks, in which they had to complete five phonological and five semantic WF trials. Meanwhile, functional transcranial Doppler sonography (fTCD) was performed (see below). All items were presented in randomized order. Participants had 30 s for each of the five items in the WF tasks. The tasks were performed in an overt setting. Further, participants had to perform the subtest *connecting numbers (*CN) of the *Nuremberg Age Inventory* (NAI, Nürnberger Altersinventar, Oswald & Fleischmann, 1999). The participants had to connect numbers from 1 to 30 in the correct order as fast as possible. The numbers were arranged arbitrarily. The time required was measured with a stopwatch. The third diagnostic tool was the subtest *figure test* (FT) of the NAI in which participants had to memorize 12 differently shaped figures and recognize them in a second step. This test assessed their non-verbal memory functions. The fourth diagnostic part consisted of assessing the participants’ mood with a multidimensional questionnaire on wellbeing (MDBF, *Mehrdimensionaler Befindlichkeits-Fragebogen*, Steyer et al., 1997). All participants completed a questionnaire assessing current mood in which they rated adjectives on a 5-point Likert scale that could later be assigned to three dimensions (good-bad, awake-tired, calm-agitated). If participants had difficulty assessing their current mood and/or answering a particular item, the experimenter helped determine the most appropriate score.

The results of the diagnostic tests underline the assumption that the third group consists of people with cognitive deficits who can be assigned to the MCI group. These are described and analyzed in the results section.

### 2.4 Functional transcranial Doppler sonography (fTCD)

**FIGURE 1:**
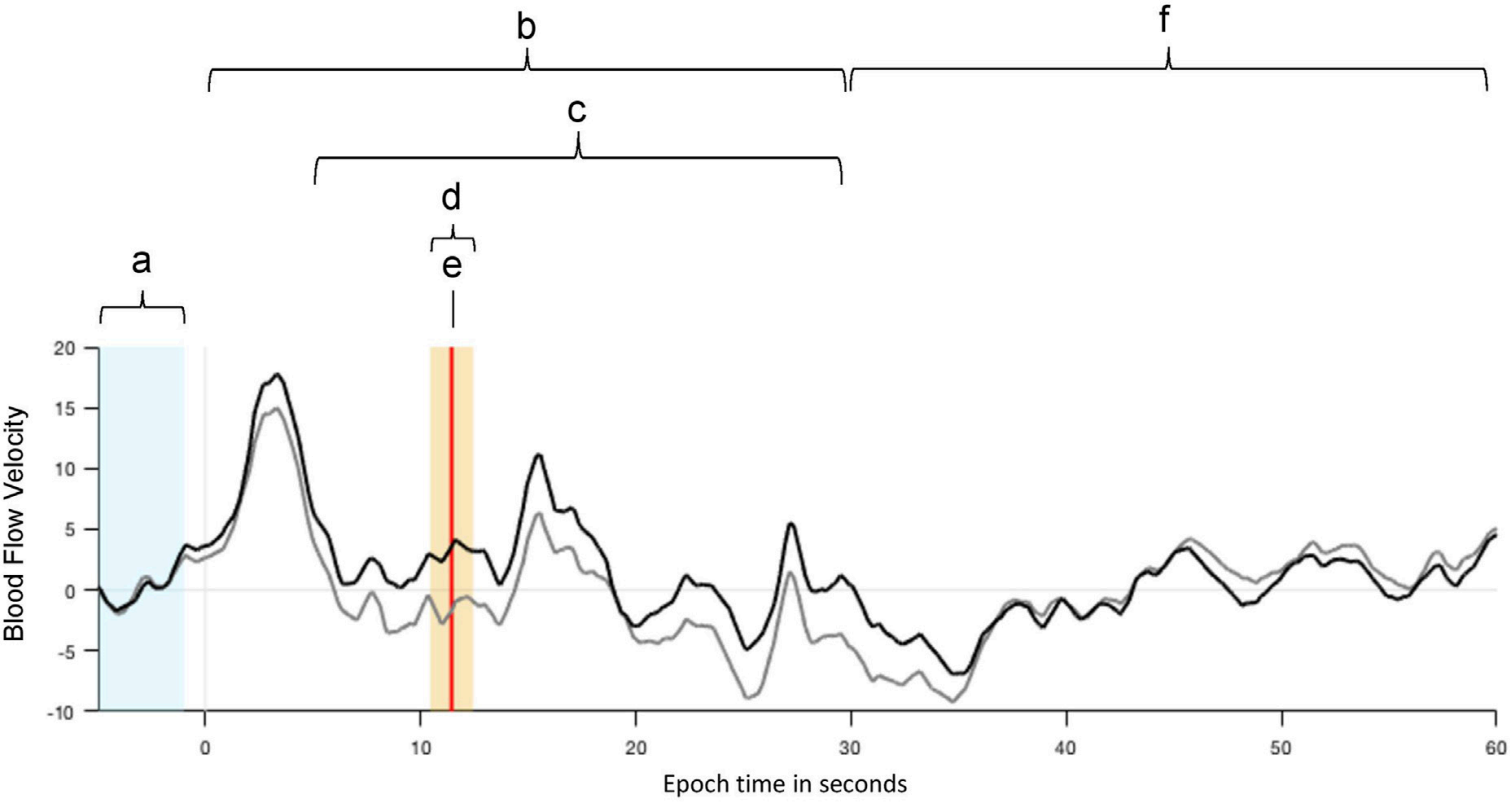
Example of mean cerebral blood flow velocities (CBFV) of the left (black) and right (grey) middle cerebral arteries (MCA) during the WF tasks. The velocity values were normalized and averaged over consecutive WF trials. **(A)** An interval of 10 s (from −15 to −5 s) before each item presentation is used as baseline (BL) for CBFV values. **(B)** The item presentation and overt word production start at 0 s and last for 30 s. **(C)** The period of interest, in which the process of neuro-metabolic coupling is considered, starts 5 s after the item presentation. **(D)** The highlighted interval of 2 s is the activation window in which the LI is calculated. **(E)** The vertical red line indicates the largest difference between the CBFV values of both MCAs. **(F)** After a subsequent relaxation phase of 30 s the next item is presented.

Functional transcranial Doppler sonography (fTCD) was performed during the phonological and semantic word fluency tasks described above to measure the participants’ patterns of language lateralization as assessed by the lateralization index (LI). This was done in session 1 as well as in session 5. Each trial comprised an initial baseline interval (Figure 1A) ranging from −15 to −5 s before the cueing tone and item presentation for later analysis, followed by a 30- second interval in which the participant generated words according to the letter or semantic category displayed on the screen (Figure 1B).

The period of interest (Figure 1C) was set to begin 5 s after item presentation to consider the process of neuro-metabolic coupling and to lasts up to 30 s relative to item presentation. The activation window (Figure 1D) itself, is the time interval with the largest event-related changes in cerebral blood flow velocities (CBFV) in the left and right middle cerebral arteries (MCAs) and includes the time point of LI calculation (Figure 1E; vertical red line), which marks the largest difference in CBFV between the left and right hemisphere after normalization. It was set to 2 s, where the LI calculation is performed by the fTCD analysis software by comparing the mean blood flow velocities in both MCAs in this time window. During the subsequent 30-s resting period (Figure 1F), the participant was instructed to rest and relax. During the last part of this resting period, seconds 55 to 59 were used as the next baseline period for the following item presentation and word generation.

For assessment of language lateralization, recorded fTCD data were analyzed using dopStep Master, which evolved from dopOSCCI, a Matlab (Mathworks, Natick, MA, United States of America) based software package (Badcock et al., 2012b). Its programming is based on the software package AVERAGE (Deppe et al., 1997), can be used with various TCD devices, and allows subtle quantitative offline analysis of Doppler flow signals. First, the channels for the left and right MCA as well as the trigger channel were set. The latter contains markers, which must be stored in the data file in order to time-lock the related activity. They are commonly sent via the parallel port before the presentation of each item. The electric trigger signals were sent from the PC to the fTCD-Computer (MultiDop T2, DWL, Sipplingen, Germany) via a customized cable connecting the PC’s DB-25 parallel port to the fTCD-Computer’s Av-in-port to mark the beginning of each trial. The computer then recorded the lateralization patterns extracted from the participants’ blood flow patterns in the left and right MCA. To avoid interference from involuntary cardiac events when examining task-related signals, the activity within a single heart cycle was averaged, which resulted in a step-like summary of the activity as opposed to the natural variations in blood flow velocity during a heartbeat. Additionally, the time span between two event markers (Figure 1, dashed line) was set to 60 s, and the range of blood flow was limited to 150 cm/s to exclude measurement of movement artifacts. Since the probe angles may differ between the two sides (Deppe et al., 2004), data from the left and right MCA are normalized to a mean of 100 using the following equation:

$$\frac{\left(100xdata\right)}{Ø\left(data\right)},$$

here data refer to the blood flow velocity values at a certain time of measurement.

### 2.5 Transcranial direct current stimulation

Transcranial direct current stimulation was administered via a battery-driven direct current stimulator (NeuroConn DC-Stimulator plus). Stimulation was delivered via two electrodes (5 × 7 cm^2^) in saline-soaked sponges (0.9% saline solution) attached to participants’ heads with rubber bands. The electrodes were placed on the head of the participants according to the international 10–20 system. The anode was placed over the crossing point Fp1–T3/Cz–F7 (part of the Broca’s area, Homan, 1988) with the long side oriented vertically, and the cathode was placed on the participants’ contralateral supraorbital region (Fp2) with the long side oriented horizontally. We applied 2 mA atDCS over the left inferior frontal gyrus (IFG) for 20 min with a fade-in and fade-out of 10 s. This resulted in a current density of 0.043 mA/cm^2^ below each electrode. Since former studies in older participants have shown beneficial effects of online stimulation (Fertonani et al., 2014), we decided to use online stimulation while participants fulfilled the task. The sham procedure was identical to the atDCS, and because the study was double-blinded and randomized, neither participants nor the experimenter knew which stimulation condition they were in. In the sham condition, the current started but was automatically ramped down after 30 s. This procedure guaranteed the participants’ blindness to the stimulation condition because it elicited a light tingling sensation on the participants’ heads that was comparable to real tDCS but did not lead to neuronal enhancement (Nitsche et al., 2008).

### 2.6 Experimental procedure

The stimulation sessions took place at Bielefeld University or at participants’ homes if this was necessary due to their immobility or mental status. All participants had the atDCS/sham sessions on similar days of the week and at a similar time of day. A schematic representation of the sequence of tasks and tests performed in each session is provided in Table 1. Session 1 was considered a diagnostic session, and session 5 was used to evaluate the intervention.

**TABLE 1:** Sequence of tasks and diagnostic instruments performed in each of the five sessions.

Session 1 diagnostics	Session 2 atDCS/sham	Session 3 atDCS/sham	Session 4 atDCS/sham	Session 5 evaluation
Interview, Handedness, Contraindications	Phonological WF	Phonological WF	Phonological WF	
-----------------------	Semantic WF	Semantic WF	Semantic WF	-----------------------
DemTect				DemTect
-----------------------				-----------------------
MDBF				MDBF
fTCD incl. phon. and sem. WF				fTCD incl. phon. and sem.WF
CN (NAI)				CN (NAI)
FT (NAI)				FT (NAI)

Abbreviations: atDCS, anodal transcranial direct current stimulation; MDBF, multidimensional questionnaire on wellbeing; ftCD, functional transcranial Doppler sonography; WF, word fluency; CN, subtest *connecting numbers* (CN) of the Nuremberg Age Inventory (NAI); FT, subtest *figure test* (FT) of the Nuremberg Age Inventory (NAI).

**FIGURE 2:**
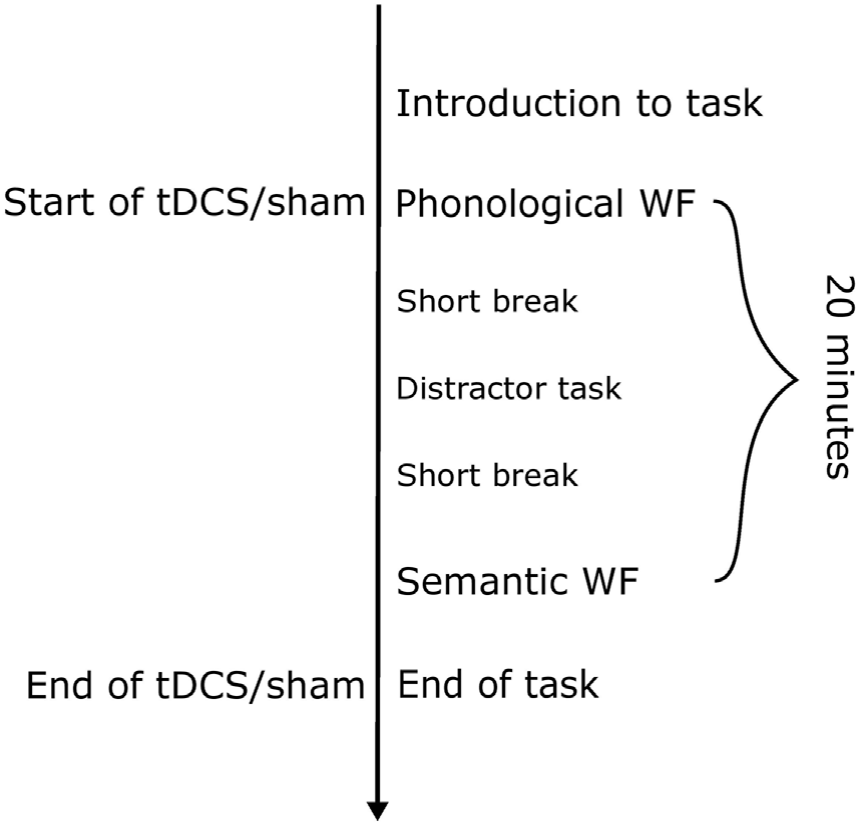
Sequence of a regular experimental session with either sham or anodal tDCS. The duration of the stimulation was 20 min. Between the two blocks of word fluency, a verbal memory task was introduced as a distractor task, to avoid having to perform the phonological and semantic WF in direct succession. This task was not evaluated. The total duration of an experimental session including the electrode application varied between 40 and 50 min.

In diagnostic session 1, participants had an interview about their personal history, including medication use and general health. Furthermore, they were tested on their handedness (modified version of the Edinburgh Handedness Inventory, Oldfield, 1971) and asked about potential contraindications related to tDCS. The diagnostic assessments were performed on the desk in front of the participant. The same diagnostic/assessment instruments were used in the first and last sessions. In sessions two to four, the atDCS/sham intervention was conducted during phonological and semantic word fluency tasks (Figure 2).

Each of the individual WF trials lasted 1 minute and was performed in an overt setting. All items were presented in randomized order for each participant. A distractor task was performed between the WF tasks so that the WF tasks did not have to be completed one right after the other. The stimuli were arranged and presented using Cogent 2000, a MATLAB-based toolbox, which was installed on a Dell-laptop PC (Windows XP). Participants sat in front of a computer monitor at a distance of 50 cm between their eyes and the screen. The letters and semantic categories for both WF tasks were visually presented in a light grey serif-free font (Helvetica size 40) on a black background on a 15″LCD monitor. The visual angle was 1,15°. Before the WF tasks were started, we conducted one exemplary phonological and semantic trial to ensure the participant understood the task. Before the experiment started, the participant was asked to rest for 1 min.

### 2.7 Statistical analysis

The data of 31 participants were included in the statistical analysis, performed with SPSS software (IBM, vers. 16) and the open-source program jamovi (The jamovi project, 2022; vers. 2.3). We grouped the participants according to age and memory performance, namely, the cut-off value of the DemTect dementia screening (18–13 = age-norm; ≤12 = less than the age norm).

To account for the differences in baseline diagnostic test scores and word fluency within and between the three groups (YG, OG, MCI), we calculated difference scores (DS) between session 5 and session 1 for diagnostic tests (DemTect, MDBF, both NAI subtests), as well as phonological and semantic word fluency. These difference scores were used as dependent variables. We calculated ANOVAs with the factors *group* (YG, OG, MCI) and *stimulation* (sham vs. atDCS). To measure changes in language lateralization index (LI) before and after the stimulation, we also calculated a difference score between session 5 and session 1 for each participant. To determine a possible relationship between WF performance, intervention, and degree of linguistic lateralization, we conducted a linear regression analysis with WF performance as the predictor and degree of language lateralization as dependent variable.

## 3 Results

### 3.1 Diagnostic session (session 1)

In the diagnostic session, the mean number of words produced in the phonological WF task was 56.8 for the YG (SD = 12.4), 52.7 for the OG (SD = 13.8), and 39.1 for the MCI group (SD = 11.3). ANOVA revealed a significant group effect in the phonological word fluency task (*F* (2,28) = 4.74, *p* = 0.017). Tukey *post hoc* tests showed that the YG produced significantly more words than the MCI group (*t* (28) = 2.92, *p* = 0.018). The OG, on the other hand, only tended to show better phonological WF than the MCI group (*t* (28) = 2.24, *p* = 0.082). The YG and OG did not differ significantly. In the semantic WF task the mean number of words produced was 62.5 for the YG (SD = 4.8), 50.3 for the OG (SD = 8.9), and 33.9 for the MCI group (SD = 8.8). Semantic word fluency also showed a significant group effect (*F* (2,28) = 28.4, *p* ≤ 0.001). Here, all groups differed significantly from each other, with the YG performing best and the MCI group performing worst. The YG was significantly different from the OG (*t* (28) = 2.99, *p* = 0.015) and the MCI (*t* (28) = 7.48, *p* ≤ 0.001). The OG also differed significantly from the MCI group (*t* (28) = 4.41, *p* ≤ 0.001).

The mean time required for the subtest *connecting numbers (*CN) of the *Nuremberg Age Inventory* (NAI, Nürnberger Altersinventar, Oswald and Fleischmann, 1999 was 20.2 s for the YG (SD = 18.2), 25.4 s for the OG (SD = 24.3), and 50.7 s for the MCI group (SD = 42.2). We found a significant group effect (*F* (2,28) = 5.8, *p* = 0.008). Tukey *post hoc* tests showed that the YG was significantly faster than the MCI group (*t* (28) = −3.16, *p* = 0.01). The OG also differed significantly from the MCI group (*t* (28) = −2.61, *p* = 0.037). The YG and OG did not differ significantly.

The mean number of memorized items in the figure test (FT) of the NAI was 10.4 for the YG (SD = 0.8), 9.7 for the OG (SD = 0.9), and 8.0 for the MCI group (SD = 1.6). This test also showed a significant group effect (*F* (2,28) = 9.17, *p* ≤ 0.001). Tukey *post hoc* tests showed that the YG recognized significantly more items than the MCI group (*t* (28) = 4.13, *p* ≤ 0.001). The OG also recognized significantly more items than the MCI group (*t* (28) = 2.97, *p* = 0.016). The YG and OG did not differ significantly.

For the mood assessment using the multidimensional questionnaire on wellbeing (MDBF, *Mehrdimensionaler Befindlichkeits-Fragebogen*, Steyer et al., 1997), none of the three dimensions differed significantly between the three groups for either of the three measured dimensions (s. 1.3 Diagnostic session (session 1).

The results of the above tests show that the MCI group had lower word fluency, lower processing speed in a trail making test for numbers, and lower recall performance in non-verbal memory compared to the other groups. All of these diagnostic tests described above were performed again in session 5 to evaluate the intervention (Table 1).

### 3.2 Word fluency and tDCS

**FIGURE 3:**
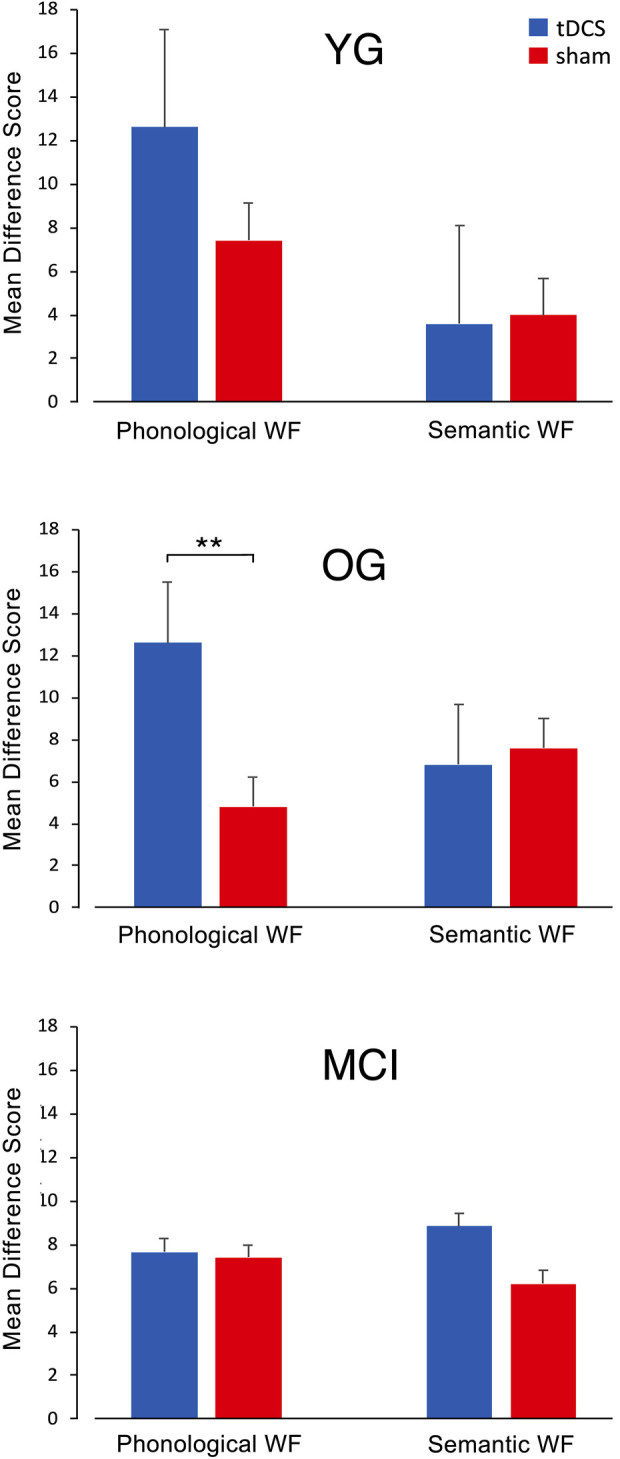
Mean difference scores (DS) for both WF tasks with atDCS and sham stimulation in the YG, OG, and MCI group. In the YG and OG, participants reached a higher learning score in the phonological WF task after anodal tDCS compared to sham stimulation, while the DS for the semantic WF task did not show much variation. In the MCI group, there were no differences in DS between stimulation conditions for the phonological WF task. For the semantic WF task, DS was higher for the anodal tDCS than for the sham treatment.

When comparing the diagnostic session and the evaluation session, both phonological and semantic WF increased in all three groups and all conditions from session 1 to session 5 (Figure 3), suggesting a general learning effect. YG participants achieved a high difference score on the phonological WF task (12.6 ± 7.71 after atDCS and 7.4 ± 2.42 after sham stimulation), indicating a significant improvement in WF performance. The difference score for the semantic WF task did not differ noticeably between stimulation conditions and was slightly higher during sham (4 ± 6.2) compared to atDCS (3.6 ± 4.8) (Figure 3). In the OG also, phonological WF was higher in session 5 after atDCS (12.6 ± 4.5) compared with sham (4.8 ± 2.8). There were no major differences in DS in the semantic WF task in both atDCS (6.8 ± 4.2) and sham (7.6 ± 7.3) (Figure 3). In the MCI group, there was little difference in DS in the phonological WF task for atDCS (7.7 ± 6.3) and for sham (7.4 ± 6.0) but a higher difference score for atDCS (8.8 ± 5.2) compared to sham (6.2 ± 2.5) during the semantic WF task (Figure 3). The sum of words produced during the five phonological and semantic WF-tasks in the diagnostic session and the evaluation session on the final day is provided in Table 2.

**TABLE 2:** Sum of words produced during the five phonological and semantic WF-tasks in the diagnostic session and the evaluation session for each participant.

Participant	Sex	Age	Stimulation condition	Phon. WF diagnostic session	Phon. WF evaluation session	Sem. WF diagnostic session	Sem. WF evaluation session
YG1	w	67	sham	53	58	63	62
YG2	w	66	sham	56	62	66	77
YG3	w	61	sham	61	68	68	78
YG4	w	65	sham	79	86	70	75
YG5	m	60	sham	41	53	67	62
YG6	w	64	tDCS	44	66	48	59
YG7	w	60	tDCS	50	69	61	64
YG8	w	63	tDCS	79	86	70	75
YG9	m	67	tDCS	57	58	54	50
YG10	m	65	tDCS	48	62	58	61
OG1	w	78	sham	83	84	67	89
OG2	w	77	sham	33	35	40	45
OG3	m	80	sham	51	59	51	53
OG4	m	76	sham	56	62	50	54
OG5	m	75	sham	61	68	56	61
OG6	w	91	tDCS	59	75	61	61
OG7	m	75	tDCS	38	51	42	50
OG8	m	70	tDCS	65	70	54	67
OG9	m	79	tDCS	37	48	35	42
OG10	m	80	tDCS	44	62	47	53
MCI1	w	75	sham	17	22	21	23
MCI2	m	85	sham	53	50	41	48
MCI3	m	85	sham	43	56	35	40
MCI4	m	78	sham	21	34	20	29
MCI5	m	79	sham	47	56	29	37
MCI6	w	100	tDCS	35	52	34	41
MCI7	w	88	tDCS	37	39	35	38
MCI8	w	74	tDCS	54	70	55	64
MCI9	m	76	tDCS	46	50	30	50
MCI10	m	75	tDCS	49	52	36	43
MCI11	m	78	tDCS	31	35	24	31

An ANOVA for difference scores on WF performance showed a significant effect of atDCS (*F* (1,25) = 4.27, *p* = 0.05) during the phonological WF task but not during semantic WF (*F* (1,25) = 0.05, *p* = 0.82). There was no significant difference between *groups* or in a two-way *group* × *stimulation* interaction. Post-hoc tests revealed significantly increased WF performance for tDCS *versus* sham for the OG (*t* = −2.95; *p* = .009).

### 3.3 Word fluency and fTCD

**FIGURE 4:**
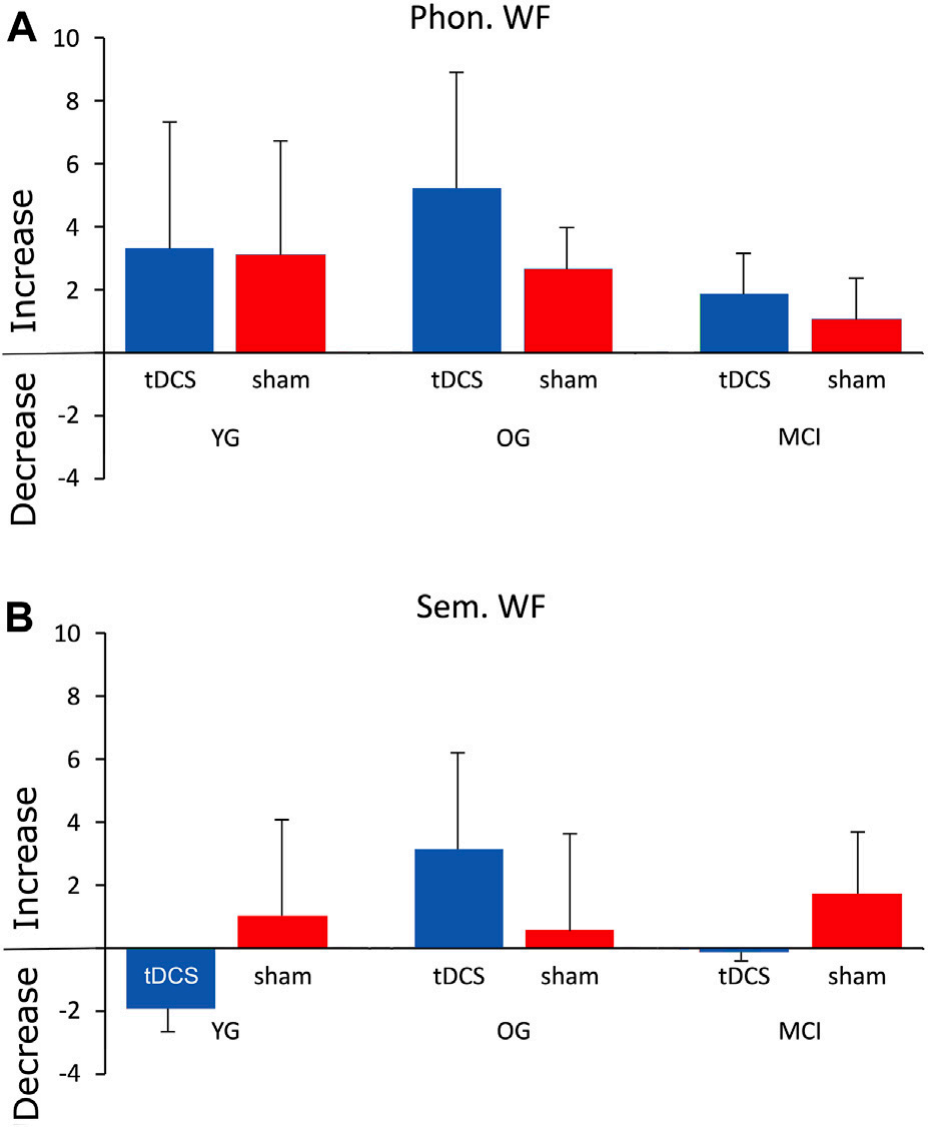
Mean difference scores of the lateralization index (subtracting the LI values of session 1 from those of session 5) for the phonological **(A)** and semantic **(B)** WF tasks.

The fTCD measurement showed left lateralization of blood flow in both word fluency tasks for all groups during the diagnostic session. The lateralization index (LI) did not differ significantly between the three groups during either phonological or semantic word fluency before treatment. LI during phonological WF increased in all groups from session 1 to session 5. This increase was evident in both stimulation conditions (Figure 4A). The LI during the semantic WF task also showed an overall increase except for the YG and MCI groups, which had lower LI values in session 5 after atDCS (Figure 4B). A generalized linear model revealed that CBF was significantly more lateralized during phonological WF in the evaluation session than in the diagnostic session (ß = 2.90, z = 3.36, *p* = .002). However, this effect did not differ between groups and was not influenced by type of stimulation. LI during the semantic WF did not change significantly from session 1 to 5.

The next step of data analysis addressed the question of whether increases in phonological word fluency were related to or predicted increases in lateralization index. Linear regression showed that the difference scores of WF performance could not significantly predict changes in the degree of language lateralization as described by the LI for the YG group during phonological (*R* ^2^ = 0.104, *p* = 0.72) or semantic WF (*R* ^2^ = 0.30, *p* = 0.35), the OG during phonological (*R* ^2^ = 0.239, *p* = 0.52) or semantic WF (*R* ^2^ = 0.258, *p* = 0.52), and the MCI group during phonological (*R* ^2^ = 0.271, *F* (2,4) = 0.744, *p* = 0.53) or semantic WF (*R* ^2^ = 0.568, *p* = 0.19).

### 3.4 Diagnostic session vs. evaluation session

Another analysis addressed the question of whether DemTect scores changed from session 1 to session 5 also determined by difference scores. There were no statistically significant changes in DemTect scores for all groups, as shown by ANOVA analysis (*F* (2,25) = 1.802, *p* = 0.17). There was also no effect of the respective stimulation condition (*F* (1,25) = 0.002, *p* = 0.96). The mean DemTect scores for the three groups and stimulation conditions are shown in Table 3. Further analyses involved the NAI subtests *connecting numbers* (CN) and *figure test* (FT), and the multidimensional questionnaire on wellbeing (MDBF). All groups became faster on the subtest *connecting numbers* (CN) from session 1 to session 5 (Table 3). This suggests that training improved processing speed specifically in the MCI group, though not statistically significant. The change was independent of whether the group actually received tDCS or sham stimulation (*F* (1,25) = 0.18, *p* = 0.68). There was also no difference between groups (*F* (2,25) = 2.12, *p* = 0.14) and no interaction between *group* and *stimulation* (*F* (2,25) = 0.46, *p* = 0.64).

**TABLE 3:** Diagnostic instruments and scores for all three groups in session 1 and session 5 under the respective stimulation conditions (sham/atDCS).

a) Mean DemTect scores (max. 18). Scores below 12 indicate a moderate memory deficit					
Group	Intervention	DemTect session 1	SD	DemTect session 5	SD
YG	sham	17.6	0.5	18.0	0
	atDCS	17.8	0.4	18.0	0
OG	sham	16.8	1.5	17.6	0.5
	atDCS	16.4	1.6	16.8	1.6
MCI	sham	10.2	1.8	11.2	1.9
	atDCS	11.0	0.8	12.6	2.3
b) Subtest *connecting numbers* (CN) for session 1 and session 5, mean time in s					
Group	Intervention	CN Session 1	SD	CN Session 5	SD
YG	sham	20.7	3.8	18.6	2.4
	atDCS	19.9	4.0	17.7	3.6
OG	sham	27.8	7.5	27.4	7.7
	atDCS	22.9	4.1	21.1	2.6
MCI	sham	62.1	44.8	50.8	28.4
	atDCS	39.3	11.5	33.7	10.6
c) Mean scores (max. 12) for the *figure test (FT)* for session 1 and session 5					
Group	Intervention	FT Session 1	SD	FT Session 5	SD
YG	sham	10.0	0.9	10.8	0.8
	atDCS	10.8	0.8	11.0	0.6
OG	sham	10.2	0.8	10.8	0.8
	atDCS	9.2	1.0	10.0	0.6
MCI	sham	8.8	1.3	10.4	0.5
	atDCS	7.2	1.8	9.5	1.0

In addition, all groups showed improvement in nonverbal memory from session 1 to session 5, as shown in the *figure test* (Table 3). However, ANOVA revealed that this change was independent of whether the group actually received atDCS or sham stimulation (*F* (1,25) = 0.04, *p* = 0.84). There was also no difference between groups (*F* (2,25) = 2.89, *p* = 0.08) and no interaction between *group* and *stimulation* (*F* (2,25) = 0.50, *p* = 0.61).

ANOVAs of the three dimensions (good-bad, awake-tired, calm-excited) of the multidimensional wellbeing questionnaire (MDBF) showed no significant results of the good-bad and calm-agitated dimensions. However, there was a significant interaction for *group* x *stimulation* for the awake-tired dimension (*F* (2,25) = 5.62, *p* ≤ 0.01), based on the fact that after atDCS the YG were less tired than the sham group; the opposite was true for the other two groups.

## 4 Discussion

### 4.1 Diagnostic session

In this study, three groups of individuals of different ages and cognitive abilities were examined. The younger healthy group (YG) differed significantly in age from the older healthy group (OG) and the individuals with MCI (MCI). On dementia screening (DemTect), the MCI group had a significantly lower score than the YG and OG. The latter two groups did not differ. Performance in phonological WF was significantly lower in the MCI group than in the YG, but only tended to be worse than in the OG. The YG and the OG did not differ. On semantic word fluency, however, the YG performed significantly better than the OG and MCI groups, and the OG was also significantly better than the MCI. On the *connecting numbers* test, both the YG and OG were faster than the MCI group, and the YG and OG did not differ. In the *figure* test, the YG and OG recognized significantly more figures than the MCI group; the YG and OG did not differ significantly. The test on wellbeing did not differ between groups.

In summary, before the atDCS/sham stimulation the MCI group differed from the other two groups mainly in lower memory performance, lower performance in nonverbal memory recognition, phonological and especially semantic WF. In the trail making test, they were significantly slower than the other two groups. The YG and OG differed only in semantic WF, with the YG retrieving significantly more items. These results fit very well with the criteria for the diagnosis of MCI according to Petersen (2016).

The fTCD measurement showed left lateralization of blood flow in both word fluency tasks for all groups during the diagnostic session which confirms frequent findings in the literature (Heinzel et al., 2013). Interestingly, the lateralization index (LI) did not differ significantly between the three groups in either task. This contradicts the assumption that in older individuals, better performance on cognitive tasks should be associated with increased bihemispheric activity compared to cognitively impaired individuals, as postulated in the HAROLD model (Cabeza, 2002), or with greater lateralization compared to MCI individuals, which has also been frequently shown in the neuroimaging literature (e.g., Yeung et al., 2016). Because of the significant differences within and between groups before the tDCS intervention, we calculated difference scores between the respective tasks and scores of the diagnostic session 1 and evaluation session 5, which were included as dependent variables in the statistics.

### 4.2 Word fluency and tDCS

The next outcome relates to the effect of tDCS/sham stimulation combined with 3 days of word fluency training on word fluency performance in evaluation session 5. Anodal tDCS (in contrast to sham stimulation) over the left inferior prefrontal cortex significantly improved WF performance on phonological but not on semantic WF tasks. This effect was present in both age groups of healthy elderly participants (YG and OG) but not in the MCI group. This is in line with Vannorsdall et al. (2016), who found better phonological WF performance after atDCS over the left Broca region, whereas semantic WF performance was better after sham stimulation. Although these differences were not statistically significant, they are hinting at the distinct neural networks activated during phonological and semantic WF, which is also supported by our results. Our results are also consistent with previous findings which showed that increased cortical perfusion in left frontal cortical regions is associated with corresponding neuronal activity during phonological WF tasks (Keilp et al., 1999; Birba et al., 2017), hence our application of atDCS over the left IFG might explain the significant increase in phonological WF performance in both groups of healthy elderly participants. It should be noted, however, that our results differ from previous research that reported significant improvement in both phonological and semantic WF tasks after stimulation over the left DLPFC (Cattaneo et al., 2011; Pereira et al., 2013). The different position of the electrodes during tDCS in these studies might offer an explanation here, so Cattaneo et al. (2011) stimulated additional not primarily task-related networks. Other differences in study design could also offer an explanation for the different results (Cattaneo et al., 2016).

Additionally, a recent study by Vonk et al. (2020) even showed that anatomical thickness in frontal and left-frontal brain structures correlates with phonological WF performance and that corresponding anatomical differences in temporal and (para-)hippocampal structures correlate with varying semantic WF performance in healthy individuals and patients with MCI or AD. Since the reduction of cortical perfusion in relevant brain areas correlates with the stage of cognitive decline in MCI and AD (Chao et al., 2010), this functional interconnection could explain the significant improvement we found in phonological but not semantic WF. Semantic WF requires a higher cognitive load and relies on other, partly non-linguistic cognitive processes (i.e., use of mental images, semantic features) that occur in extensive neural networks of the temporal and parietal cortex (Keilp et al., 1999; Vonk et al., 2020). These were not directly stimulated by left inferior atDCS. However, results of previous studies also show a significant effect of atDCS over the left IFG on semantic WF performance in healthy elderly participants (Meinzer et al., 2013; 2015) and in patients with dementia-related cognitive decline (Penolazzi et al., 2013; Smirni et al., 2021), but their experimental task procedure and the associated word retrieval of the subjects were very different and not very comparable to our tasks.

In participants with MCI, atDCS did not induce an increase in WF compared with sham stimulation. One reason for this could be the selection of the position of the stimulation electrodes, which is a possible limitation of our study. It is likely that brain regions particularly affected by neurodegenerative processes have reduced overall neuronal activity and thus cognition can only be improved by interventions such as atDCS under certain conditions. It is well-known that tDCS is only effective when neurons in the stimulated brain regions are active. As a result, it is possible that the neural processes necessary to increase WF in persons with MCI cannot be modulated by stimulation lasting only 3 days. For instance, recent findings indicate that improvements in phonological and semantic WF were observed in MCI patients after 20 days of atDCS (Fileccia et al., 2019). Word recognition was also significantly accelerated in MCI patients after a single stimulation of the temporal cortex (Balduin-Phillipps et al., 2021). It is possible that stimulation of the temporal cortex could lead to better outcomes in MCI, as also suggested by Chen et al. (2022). Moreover, cathodal stimulation of the right DLPFC improved WF in mildly affected AD patients by supporting left hemisphere networks through short-term inhibition (Smirni et al., 2021). From this, one could assume that the same stimulation conditions do not apply to MCI patients and healthy elderly and therefore do not lead to comparable results. Consequently, stimulation conditions would need to be adapted to a person’s neuronal and cognitive status. Possibly, this assumption is also supported by the fact that only in the MCI group semantic WF after tDCS is slightly increased than in the sham group. Thus, they react differently than the healthy subjects. Another limitation of our study could be that WF was not measured during tDCS, but in session 5, 1 day after the last stimulation. Improvements in WF may not last as long in MCI patients and thus can only be observed online during the task (Chen et al., 2022). In our approach, measuring WF performance in the last session 1 day after the last anodal stimulation might be too late to detect associated improvements compared to sham stimulation − especially with only 3 days of stimulation.

### 4.3 Word fluency and fTCD

A further finding concerned the language lateralization index (LI) during the phonological and the semantic WF tasks, which was measured by functional transcranial Doppler sonography (fTCD). The lateralization index (LI) was left lateralized in both word fluency tasks for all groups during the diagnostic session, but did not differ significantly among the three groups. In phonological WF, blood flow lateralization was significantly higher in session 5 than in session 1, in all groups. Although lateralization was higher overall, particularly in OG after atDCS, this result was also not significant. The reason for the lack of significance was, presumably, the high variance within groups due to difficulties in measuring LI in some subjects. Moreover, it is possible that a significant effect would have been found if LI had been measured immediately after stimulation. Since an increasing degree of language lateralization in phonological WF was observed in all groups, it could be speculated that WF training produced greater lateralization associated with better performance (Yeung et al., 2016). This was true for MCI group, although the increase in the degree of language lateralization was smaller here than in the YG or OG. For semantic word fluency, lateralization increased similarly only in the OG. The YG even showed a decrease in lateralization after tDCS. These changes were also not significant. The finding that there were even negative LI difference scores in the YG and the MCI group for the semantic WF task might be a result of the fact that 1) semantic WF performance could not be adequately targeted by anodal stimulation of the left frontal IFG and 2) an increase in semantic WF performance, contrary to phonological, results in a higher activation in posterior neural networks (Gourovitch et al., 2000; Kitabayashi et al., 2001; Birn et al., 2010).

Another question in this study was whether the increase in WF performance was related to language lateralization. Here, the question was whether the significant increase in phonological WF after tDCS correlated with the significant increase in LI, as postulated by Yeung et al. (2016), or with a more bihemispheric pattern, as postulated in the HAROLD model (Cabeza, 2002). Neither phonological nor semantic WF performance changes could predict the degree of left lateralization, consistent with the results of Lust et al. (2011). Whether this resulted from the small number of subjects and thus high variability in WF performance or LI, or from the difficulty in measuring CBF in some subjects, could not be clarified by this study. In any case, the number of subjects per group could be another limiting factor of the study. Moreover, examining the correlation between a broader spectrum of cognitive functions (i.e., overall scores during dementia screenings) and the direction and degree of language lateralization might be useful for understanding neurophysiological mechanisms in neurodegenerative diseases.

### 4.4 Diagnostic session vs. evaluation session

The behavioral tests (DemTect, connecting numbers, figure test) did not show changes in any group as a function of atDCS between the first and the last session. However, scores in the DemTect increased for all groups, indicating a general learning effect. This was also true for the results of the NAI subtest *connecting numbers* (CN), which showed a general reduction in the time needed for the task in all three groups. However, the improvement was comparatively more pronounced in the MCI group than in the YG and OG groups (Table 3b).

Regarding participants’ mood tested via MDBF, the only change as a function of atDCS was that the YG was significantly less tired than the sham group after the 3 days of stimulation; the opposite was true for the other two groups. A possible explanation for this finding might be that the YG are more physically active compared to OG and MCI, which is associated with better psychosocial wellbeing (Finkenzeller et al., 2019) and therefore might affect specific dimensions of mood assessment. It is possible that the intervention with atDCS enhanced these differences.

### 4.5 Conclusion

In summary, unlike phonological WF, semantic WF and all other cognitive tests showed no significant change after atDCS for three consecutive days in healthy elderly and elderly with MCI. This implies that stimulation of the IFG is specific for improving phonological WF, at least in healthy elderly. Left lateralization was not significantly affected by atDCS but showed significantly higher values after 3 days of training. In future studies, blood flow should be measured during atDCS to verify whether lateralization changes more online than offline. To improve semantic WF, a different electrode configuration and/or more frequent stimulation would probably need to be targeted. The lack of improvement in phonological and semantic WF in the MCI group suggests that experimental stimulation parameters likely need to be adjusted to a person’s neuronal and cognitive status. Training of WF and other cognitive functions in MCI is certainly useful, but needs to be additionally supported by interventions such as individualized atDCS. Further research with larger samples (Minarik et al., 2016) and altered stimulation parameters is needed to investigate whether this can produce more successful results in MCI patients and patients with more severe dementias (e.g., Alzheimer’s disease).

## Data Availability

The raw data supporting the conclusion of this article will be made available by the authors, without undue reservation.
